# Overexpression of Arabidopsis *FLOWERING LOCUS T* (*FT*) gene improves floral development in cassava (*Manihot esculenta*, Crantz)

**DOI:** 10.1371/journal.pone.0181460

**Published:** 2017-07-28

**Authors:** O. Sarah Adeyemo, Paul Chavarriaga, Joe Tohme, Martin Fregene, Seth J. Davis, Tim L. Setter

**Affiliations:** 1 Soil and Crop Sciences Section, School of Integrative Plant Science, Cornell University, Ithaca, New York, United States of America; 2 Department of Plant Developmental Biology, Max Planck Institute for Plant Breeding Research, Cologne, Germany; 3 International Center for Tropical Agriculture (CIAT), Cali, Colombia; Shanghai Institutes for Biological Sciences, CHINA

## Abstract

Cassava is a tropical storage-root crop that serves as a worldwide source of staple food for over 800 million people. Flowering is one of the most important breeding challenges in cassava because in most lines flowering is late and non-synchronized, and flower production is sparse. The *FLOWERING LOCUS T* (*FT*) gene is pivotal for floral induction in all examined angiosperms. The objective of the current work was to determine the potential roles of the *FT* signaling system in cassava. The *Arabidopsis thaliana FT* gene (atFT) was transformed into the cassava cultivar 60444 through Agrobacterium-mediated transformation and was found to be overexpressed constitutively. *FT* overexpression hastened flower initiation and associated fork-type branching, indicating that cassava has the necessary signaling factors to interact with and respond to the atFT gene product. In addition, overexpression stimulated lateral branching, increased the prolificacy of flower production and extended the longevity of flower development. While *FT* homologs in some plant species stimulate development of vegetative storage organs, atFT inhibited storage-root development and decreased root harvest index in cassava. These findings collectively contribute to our understanding of flower development in cassava and have the potential for applications in breeding.

## Introduction

In storage-root crops such as cassava (*Manihot esculenta*, Crantz), research on flowering has received relatively little attention. This is partially because floral, fruit and seed organs are not the harvested parts of the plant. However, in cassava breeding, delayed and non-synchronous flowering is a major impediment for crossing selected lines [1, 2]. Many elite lines with desirable agronomic traits including high yield of storage-roots and erect non-branched shoot architecture, are difficult to use as parents because their flowering is late and sparse [2]. Understanding the factors that regulate flowering in cassava would be valuable to facilitate progress in breeding programs. Furthermore, if the regulatory system were better understood, it might be possible to develop methods for hastening floral initiation so that desirable alleles, which are otherwise “locked up” in parents with poor flowering, will become available. Controllable flower induction could help breeders make more rapid progress by enabling earlier crosses, thereby shortening the breeding cycle [3].

*Flowering Locus T (FT)* in Arabidopsis (atFT) is now recognized as the key component whose expression is regulated by upstream signaling components that perceive photoperiod, vernalization (cool temperatures of winter), and other factors in leaves [4]. The translated protein of atFT is the flowering stimulus which interacts with signaling factors in the apical meristem [5–7]. The “florigenic” signal is the translated protein of the FT gene that is transported via phloem from leaves to the apical meristem where it causes the switch from vegetative to reproductive development [8].

The role of the *FT* gene in flower induction has been established in many species of angiosperms, including all examined dicots and monocots [4–6, 9]. There is evidence that *FT* signaling plays a role in photoperiodic and developmental regulation in species closely related to cassava. In Barbados nut (*Jatropha curcas*), which like cassava is in the Euphorbiaceae family, an *FT* homolog is primarily expressed in the reproductive organs and is thought to play a role in flower induction [10, 11]. In leafy spurge (*Euphorbia esula*), long photoperiods (16 h light) stimulates accumulation of *FT* homologs in a diurnal manner consistent with flower induction. On the other hand, under long days and cooling temperatures, *FT* expression is down regulated, and *DAM* (*DORMANCY ASSOCIATED MADS BOX*) is up-regulated, a response associated with induction of overwintering bud dormancy [12]. Similarly, Böhlenius et al. [13] demonstrated that in poplar (*Populus trichocarpa*), which is in the Salicaceae family, closely related to Euphorbiaceae, flowering is induced by long days and corresponding induction of diurnal expression of *PtFT1*, while shortening days induce growth cessation and vegetative bud set in advance of winter.

Overexpression of transgenic atFT has been shown to induce early flowering in woody plants with long juvenile phases such as blueberry (*Vaccinium corymbosum* L.) [14] and eucalyptus (*Eucalyptus grandis* x *Eucalyptus urophylla*) [15]. Also, overexpression of an FT homolog from *Jatropha curcas* was constitutively overexpressed with CaMV-35S in *J*. *curcas* to demonstrate enhanced flowering [10], and FT overexpression in various paired species has accelerated flowering in apple (*Malus* spp.) [16, 17], and poplar (*Populus trichocarpa*) [18]. Given the effectiveness of this approach, it has been suggested that *FT* overexpression could be used to hasten flowering in breeding programs [15, 18–20]. In cassava, breeding might benefit if genotypes with abundant production of the FT signal were used as understocks in grafting such that breeding lines would not be stably transformed [21].

The objective of the current study was to overexpress the Arabidopsis *FT* gene in cassava and determine whether the cassava signaling system interacts with and responds to the Arabidopsis *FT* with earlier flower induction. Our findings indicate that cassava responds to overexpression of Arabidopsis *FT* with extremely early flowering. *FT* overexpression also substantially increased the number of flowers produced and lengthened the duration of cassava flowering such that abundant mature flowers were obtained. These studies improve our understanding of flowering regulation in cassava and indicate the potential for application in breeding programs.

## Methods and materials

### Molecular cloning and plant transformation

**Fig 1:**
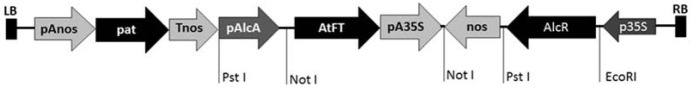
Schematic representation of the transformation vector. *Arabidopsis FT* cDNA was inserted into the construct through Gateway cloning. pAnos, nopaline synthase polyadenylation signal; pat, phosphinothricin acetyltransferase; Tnos, terminator of nopaline synthase; pAlcA, promoter of alcohol dehydrogenase I (Adh-I) encoded by the *alc*A gene; *FT* cDNA, cDNA of Flowering Locus (FT) gene; pA35S, polyadenylation sequence of Cauliflower mosaic virus 35S gene; nos, nopaline synthase terminator; ALCR, transcriptional factor which binds to *AlcA promoter*; p35S, Cauliflower Mosaic Virus 35S promoter; LB, left border; RB, right border.

The ORF of *FT* (At1g65480) was amplified by PCR, using GATEWAY^TM^ compatible primers (FTGWFW- GGGGACAAGTTTGTACAAAAAAGCAGGCTCCATGTCTATAAATATAAGAGACCCTC and FTGWRV- GGGGACCACTTTGTACAAGAAAGCTGGGTCTAAAGTCTTCTTCCTCCGCAGCCA). The resultant attB-FT-PCR product was cloned into the pDONR207 vector (Thermo Fischer Scientific) using BP Clonase, and the sequence-validated insert from FT-pENTRY clone was subcloned into the pNew-Mik1-antisense GATEWAY-compatible vector (Destination vector; Bekir Ülker, MPIPZ), using LR Clonase (Gateway; Invitrogen). The plant expression vector created expresses *FT*-cDNA under the control of a CaMV35S promoter and an ethanol inducible system (Fig 1). This plasmid was introduced into Agrobacterium ABI [22] by electroporation and transferred to friable embryogenic callus (FECs) of cassava genotype 60444 by the Agrobacterium-mediated transfer method, as described by Gonzalez et al. [23], with modifications that promote transformation in several cassava varieties [24]. For these studies transformants from independent transformation events, designated FT-02, FT-11, FT-13, FT-17 and a non-transformed control, 60444 are reported. To confirm that the transgene was incorporated into cassava according to expectations, we performed a PCR of genomic DNA that shows the amplified product of atFT gene in the four transformants, the untransformed cassava, and in Arabidopsis control DNA (S1 Fig).

### Plant materials and growth conditions

The *in vitro-*maintained putative transgenic cassava plantlets which are maintained at CIAT (http://genebank.ciat.cgiar.org) were grown from subcultured stem segments for about 4 weeks to about the 3-leaf stage [25]. The plantlets were carefully removed from test tubes, agar was washed off, and planted in sterile peat/vermiculite/pearlite rooting medium. The plantlets were covered to maintain a humid environment with inverted clear polystyrene cups. After about one week cups were replaced with polyethylene bags, which were progressively punctured more and more over about three weeks to gradually lower humidity and promote root growth. Plantlets were carefully watered, as needed. They were then transferred to the green house where they were maintained with temperature controlled at 30^°^C (day)/25°C (night), under long days (14h light and 10h dark) with natural illumination supplemented with about 150 μmol m^-2^ s^-1^ of photosynthetically active radiation (400 to 700 nm) from metal halide lamps. These plants were propagated into four batches of plants which were used for subsequent studies of their architecture and expression of the introduced *FT* gene. Three batches were grown directly from *in vitro* plantlets; ethanol treatments were initiated at 4 months after planting (MAP) (batch 1 and 2) or 3 MAP (batch 4). Batch 3 was established from stem cuttings taken from batch 1, and ethanol treatments were initiated at 3 MAP. In the *FT*-transformed lines in batches 2, 3, and 4, branch shoots and developing flowers were pruned off as soon as they appeared to create a more uniform plant architecture consisting of a single central stem. When ethanol treatments were initiated no further pruning was conducted. Plants in each batch were randomly assigned ethanol or water drench treatments. Each genotype by treatment combination, Batches 1, 2, 3, and 4 had 1, 1, 2, and 3 within-batch replicate plants, respectively. Ethanol/Water treatments consisted of twice weekly drenching of the soil with 500 mL of 1% (v/v) of ethanol/water over five weeks. Leaf tissue was sampled from the second most recently matured leaf on each plant, 24 hours after the fourth treatment. Leaf tissue was immediately frozen in liquid N_2_, and transferred to -80°C for storage until RNA extraction.

### Gene expression studies

Tissue was ground to powder with mortar and pestle under liquid N_2_. Total RNA was extracted using a modified CTAB protocol reported by Monger et al. [26] and quantified by absorption at 260 nm (NanoDrop ND-1000, Wilmington, DE, USA). Two μg of the total RNA was used for cDNA synthesis. Prior to the synthesis, RNA was treated with 10U/μl DNase I (Roche) with DNase 1 Buffer and incubated at 37°C for 30 min to remove any residual genomic DNA. cDNA synthesis was performed by qScript cDNA Supermix (Quanta) and Superscript III First strand synthesis supermix (Invitrogen), following the manufacturer’s instructions. Quantitative Real Time PCR was performed using PerfeCTa^TM^ SYBR^®^ Green FastMix^TM^ (Quanta) in a Bio-Rad CFX96^TM^ Real-Time System, C1000^TM^ Thermal Cycler. Primers for cassava 18S RNA were 18SF- ATG ATA CGA CGG ATC GC and 18SR- CTT GGA TGT GGT AGC CGT TT and for ubiquitin (UBQ10F-GCA ACT TGA GGA TGG CCG AA and UBQ10R-CTC CCC TCA AAC GCA GAA CA); these genes were used as internal controls. The Real-time quantitative PCR was repeated with 7 biological replicates (1 each from batch 1 and 2; 2 from batch 3; and 3 from batch 4), and each sample was assayed in duplicate using primers **AtFTL2-** AAG TCC TAG CAA CCC TCA CCT C and **AtFTR2-** CAC CCT GGT GCA TAC ACT GTT. Data for the number of PCR cycles to reach the threshold (Ct), were normalized for 18S Ct values in each specimen by subtraction (ΔCt). Values were also normalized for each specimen’s UBQ Ct value, and the 18S and UBQ normalized ΔCt values were averaged. These ΔCt values were further normalized against the 60444 water-treated controls in each batch (ΔΔCt) and interpreted as normalized fold expression (log_2_) assuming a PCR efficiency of 1.0. When the data were plotted on this log_2_ scale they were normally distributed, a requirement for statistical analysis. These Ct values were subjected to analysis of variance (ANOVA), as described below.

### Flowering traits

In cassava, flowering is associated with fork-type branching which occurs via outgrowth of axillary meristems subtending the shoot apical meristem [27]. After the first fork, two to four second-tier shoots develop and each of them initiates flowers at their shoot apexes (second tier flowers). Third and subsequent tiers of flowering develop similarly. Flowering traits were recorded weekly in Batches 3 and 4, which had 2 and 3 biological replicates each, respectively to determine: a) date of flower or inflorescence appearance, b) number of flowers that exceeded a 2-mm diameter threshold size, and c) initial date of flower (and/or inflorescence) senescence. From these weekly records, the total number of flowers at each forking tier were calculated.

### Plant growth traits

At 4.5 months after plant establishment in soil, plant height was measured and plants from Batches 1 to 4 were harvested. The number of shoot nodes between the soil surface and first forks, between the first-tier and second-tier forks, and between the second- and third-tier forks were counted. Lateral branches which formed in the axils of leaves on the main stem were counted and the presence/absence of flowering at their shoot apexes was recorded. Storage-roots were excavated from soil and counted. Storage-roots and above-ground plant parts were dried at 70°C to a constant weight, and weighed. Fibrous roots were not recovered. Harvest index (HI) was calculated as: HI = (storage-root dry mass)/[(storage-root dry mass) + (above-ground dry mass)].

### Statistical analysis

Gene expression, flowering, and growth traits were subjected to analysis of variance (ANOVA) using a model for determining effects due to ethanol drench treatment (T), effects due to *FT* overexpression genotype (G), effects due to batches (block) (B), and effects due to interaction of T×G. Each trait was analyzed using the linear model in R (version 3.1.1, R Foundation for Statistical Computing, http://www.r-project.org/).

## Results

### Cassava transgenic lines over-express Arabidopsis FT

**Fig 2:**
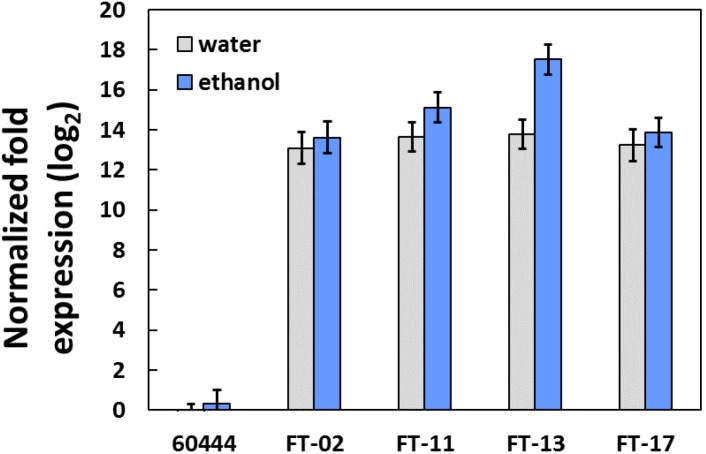
Expression of Arabidopsis *FT* gene in cassava. The qRT-PCR results were obtained from four biological replicates and two technical replicates for each sample. 60444 represents the non‐transformed wildtype line and FT-02, FT-11, FT-13 and FT-17 represent the four independent transformants. The levels of detected amplification were normalized using 18S and Ubiquitin as reference genes. The expression cassette had an ethanol‐inducible promoter. In each case, potted cassava transgenic plants were either watered normally (H_2_O), or the soil was drenched with 1% (v/v) ethanol for two weeks before leaves were harvested and analyzed.

The construct used for transformation of cassava line 60444 contained an ethanol-inducible promoter upstream of the Arabidopsis *FT* (atFT) gene (Fig 1). The transgenic events generated from the agrobacterium-mediated transfer were numbered from 1 to 22. Of these initial independent transformation events, many of them were weak and slow growing with many flowers relative to leaves such that only four of them survived after several months in culture. For this manuscript, the four surviving transformants were used. The Arabidopsis-derived *FT* transcript, expressed on a logarithmic scale such that data are normally distributed, was abundant in all the transgenic cassava lines (FT-02, FT-11, FT-13 and FT-17), while it was not detected in the untransformed control (60444) (Fig 2). Contrary to expectation, in most of the transformed lines (FT-02, FT-11 and FT-17), ethanol treatment did not further enhance expression in leaf tissue (Fig 2). Only in the transgenic line FT-13 did ethanol significantly (P ≤ 0.05) increase expression of the FT transcript in comparison to its water treated counterpart. The wild type, untransformed control, had no detectable atFT message with or without ethanol treatment.

### The Arabidopsis FT gene hastens flowering in cassava

**Fig 3:**
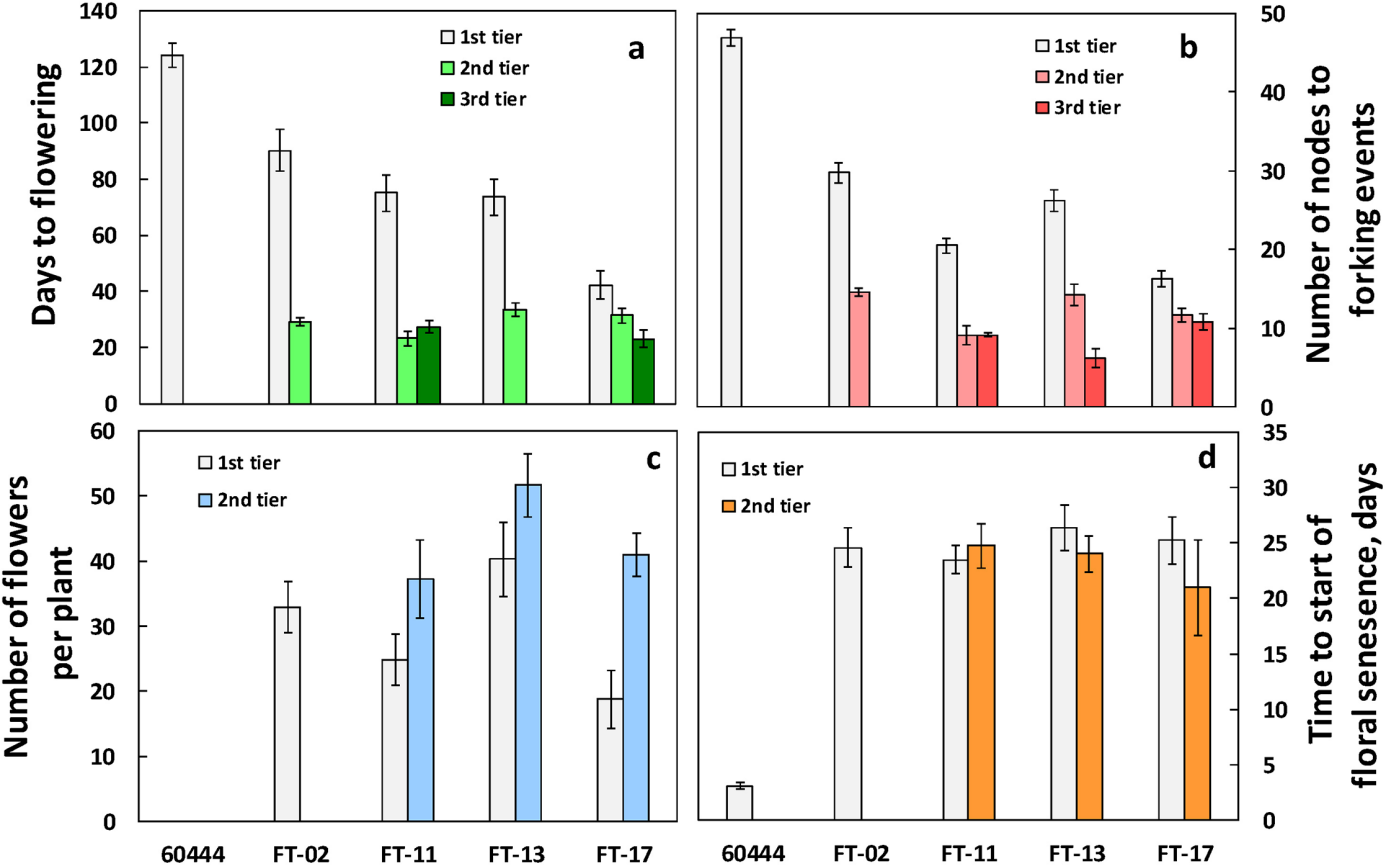
Flowering traits in non‐transformed wildtype line (60444) and in the four independent transformants. (a) Flowering time in days from establishment in soil to flowering at the 1st, 2nd, and 3rd tier of flowering, as defined by fork-type branching at the apical meristems. (b) Number of shoot nodes to forking events where inflorescences develop. The number of nodes between the soil surface and the first fork, between the first-tier and second-tier forks, and between the second- and third-tier forks. (c) Number of flowers per tier, per plant. (d) Time to start of floral and/or inflorescence senescence. Floral traits were recorded weekly to determine the date of inflorescence appearance, and initial date of floral senescence. The total number of days from flower appearance to start of inflorescence and/or flower senescence was calculated from these weekly records. Shown are the means ± SEM.

**Fig 4:**
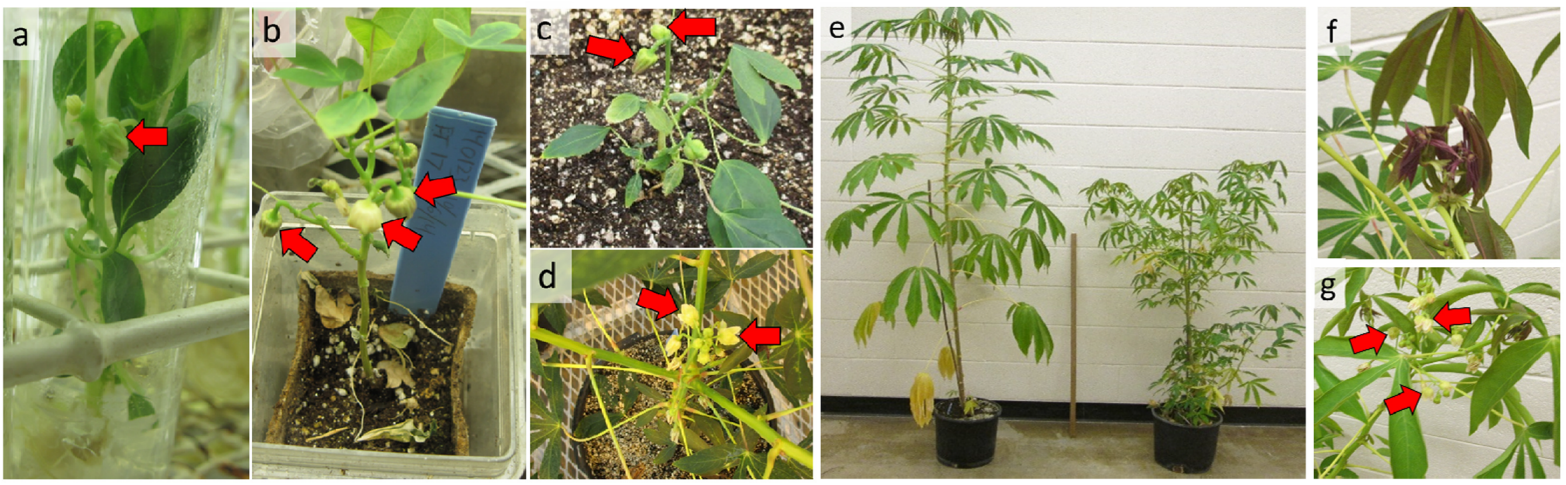
Transformed and non-transformed plants at various stages of floral development. (a): FT-17 transgenic plant at 2 months *in vitro*. (b and c): FT-17 transgenic plantlet at one month after transfer from *in vitro* to culture box and soil respectively. (d): Advanced stage transgenic plants flowering at 3 months. (e): Non-transformed (left) vs. transformed (right) plants at 5 months old. (f and g): Close up view of the apical region of 5-month old non-transformed (f) and transformed (g) plants, respectively. Arrows indicate flowers.

Due to our interest in hastening reproductive timing, we evaluated the timing of flower appearance in the atFT transformed lines throughout their development. The untransformed line, 60444, displayed its first fork-type branching and corresponding floral stalks at 120 days after transplanting (Fig 3). In contrast, the transformed lines first formed flowers while the plants were still at the seedling stage (Fig 4A–4D), and had numerous branching events associated with flowering. Indeed, flowers were observed during *in vitro* growth before transplanting to soil (Fig 4A).

To create a set of atFT-transformed material that would be well matched in size and initial architecture so that the potential effects of ethanol-induced expression of atFT could be tested, we pruned away flowers and branches so that initially each plant would have just one main stem. These plants were then allowed to form fork-type branches and flowers in the absence of ethanol treatment. The atFT plants treated in this way flowered at about 75 d after transplanting (Fig 3). Drenching with ethanol to induce the expression of atFT did not significantly (P≤ 0.05) hasten the second and subsequent forking and flowering events (S2 Fig). Given the absence of effect of ethanol treatment, the data on flowering phenology are presented as the overall average for treatments with and without ethanol treatment. Corresponding data for each of the ethanol and control treatments are shown in (S2–S8 Figs). Second fork-type branches and associated flowering occurred at only 25 to 32 days after the first flush of flowers, and in two of the transformed lines (FT-11 and FT-17) a third tier of flowering occurred about 28 days after the second tier (Fig 3). The four transformed lines did not differ significantly in the time interval between the first and second flowering events; however, FT-02 and FT-13 did not advance to a third tier of flowering during the observation period. Another indication of the timing of floral initiation events is the number of nodes between forking. Overexpression of *FT* had similar effects on the number of nodes between fork-type branches (Fig 3B). In atFT13, despite having an increased expression of *FT* in response to ethanol treatment, flowering was not further hastened between the first and second or subsequent forking and associated flowering events (S2 Fig).

### Overexpression of Arabidopsis FT in cassava results in profuse flowering

While expression of atFT has been observed to hasten flowering time in many plant species, an additional effect in the current study was sustained flower development and greater longevity of flowers (Fig 3D). We counted the number of flowers at each tier (fork) in each plant (Fig 3C) and also observed the length of time they continued to develop in each tier before they began senescing (Fig 3D). In the non-transformed controls, plants forked, and developed an inflorescence stalk with immature flower buds less than the 3-mm minimum for counting that wilted and senesced within 2–3 days (Fig 3D). In the transgenic lines, however, flower development at each tier was sustained such that more flowers were formed, and flowers continued development through anthesis rather than aborting development and senescing, as was observed in the untransformed 60444 control. Flower development traits differed in the four transformed lines corresponding to the earliness of floral initiation. The average number of flowers in FT-02, the latest to flower, was 33, followed by that of line FT-11 with 55 flowers (summed over the first and second tier). FT-13 and FT-17, the earliest lines to flower, had 77 and 60 flowers (summed over all tiers), respectively. Although third-tier flowering had commenced during the observation period in FT-11 and FT-17 (Fig 3A and 3B), flowering at tier 3 was not advanced sufficiently to obtain flower counts in any of the genotypes (Fig 3C). The longevity of the flowers produced by the over-expressing lines was also affected. Plants overexpressing atFT plants produced numerous female and male flowers, which developed fully and reached anthesis. Whereas nontransformed controls began senescing at 3 days after appearance, flower development in the transformed lines continued for almost a month and did not begin senescing until 25 to 27 days on the first tier, and 21 to 25 days on the second tier (Fig 3D).

**Fig 5:**
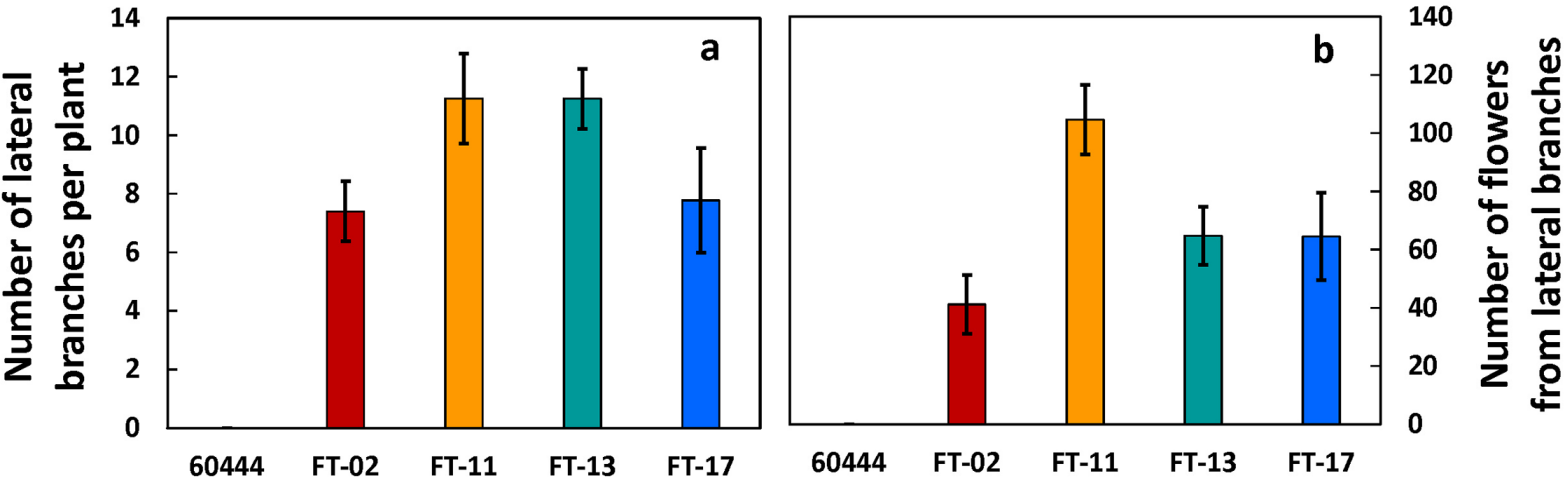
Lateral branch development in the axils of leaves on the main stem. Lateral branches and flowers that formed in fork-type branches at the apex of these lateral branches were counted in the non‐transformed wildtype line (60444) and in the four independent transformants. (a) Number of lateral branches per plant. (b) Total number of flowers on lateral branches. Shown are the means ± SEM.

In addition to fork-type branching by outgrowth of axillary meristems subtending the shoot apical meristem, atFT overexpression stimulated the outgrowth of lateral branches in the axils of leaves (Fig 5A), all of which forked at their apexes and formed flowers during the observation period (Fig 5B). Whereas the non-transformed control did not form lateral branches from axillary bud outgrowth, the transformed lines developed between seven (FT-02 and FT-17) and eleven (FT-11 and FT-13) lateral branches (Fig 5).

### Yield characters are hampered in cassava over-expressing FT gene

**Fig 6:**
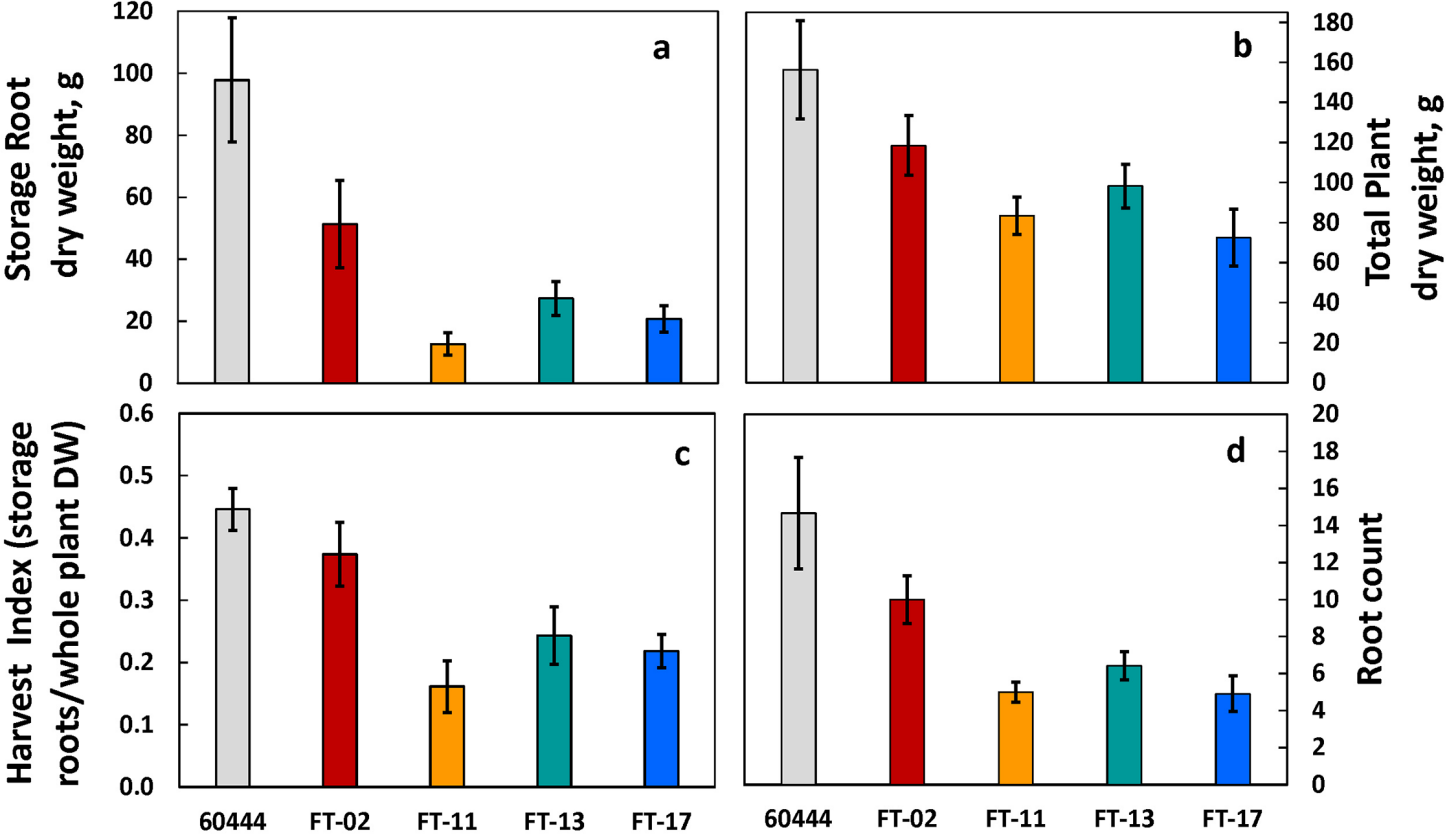
Root and shoot production in non‐transformed wildtype (60444) and the four independent transformants at harvest. (a) Storage-root dry weight; (b) total plant dry weight; (c) harvest index (HI), calculated as HI = (storage-root dry mass)/ [(storage-root dry mass) + (above-ground dry mass)]; (d) number of storage-roots. Shown are the means ± SEM.

Storage-root dry weight, total plant dry biomass, harvest index and root count of the transgenic plants as well as the control, were all measured as a function of crop yield and productivity. In general, the *FT* transformants were shorter (S8 Fig), had less storage-root production (Fig 6A), less total plant dry biomass (Fig 6B), a lower harvest index (Fig 6C), and root count than in the non-transformed wildtype (Fig 6D). The non-transformed line (60444) had the highest amount of storage-root production and harvest index, followed by FT-02, the intermediate line; and the three lines with the best flowering, FT-11, FT-13 and FT-17 had the lowest storage-root weights and harvest index (Fig 6A and 6C).

## Discussion

Delayed and scarce flowering in cassava has been a long-standing hurdle faced by conventional breeders, molecular biologists and geneticists in their attempts to cross desirable parents for improvement of cassava [1, 3]. The difficulties arising from the flowering biology of cassava have limited the development of inbred and hybrid lines for use in cassava genetic enhancement and reduced the potential impact of genomic selection [1, 3]. In the current work, we overexpressed Arabidopsis *FT* in cassava cultivar 60444, which is normally late flowering [28]. Expression was driven with the ALCR/alcA promotor system, which is designed to be ethanol inducible [29] and has been used as such in several plant species [30–34]. We applied ethanol as a soil drench, which is expected to result in root uptake of ethanol and its delivery via the transpiration stream to leaves where expression is induced, as others have shown [34]. However, in this study, leaf expression of the atFT transcript was already high in the controls (water drench treatments) of all four independent transformation events, and was not increased further by ethanol treatment (P≤0.05) except in the FT-13 line (Fig 2). In addition to expression in leaves, we also observed expression of a similar magnitude in flower buds and tissue of the apical region including unexpanded leaves and shoot meristem in transformed plants, whereas the untransformed cassava plants had insignificant atFT expression (S9 Fig). Furthermore, in the transgenic lines the plants given water versus ethanol treatment did not differ significantly for flower development traits (S2, S3 and S4 Figs). Apparently the promoter gave constitutive overexpression in the absence of added ethanol. It is possible that cassava tissues produced sufficient ethanol to drive expression from the promoter. Studies have shown that hypoxia can develop in internal plant tissues such as vasculature [35], which might have elicited ethanol production in cells of internal tissue such as the phloem. A similar finding of constitutive expression was found with the ALCR/alcA promoter system in tobacco tissue cultures [33].

The current study showed that Arabidopsis *FT* (atFT) overexpression substantially reduced the time to flowering (Fig 3) to the extent that flowering occurred in seedling plants grown *in vitro* (Fig 4). This finding is in agreement with earlier work in other species where it has been established that the *FT* gene is a key signaling factor whose expression is regulated by photoperiod and other environmental factors, and its translated protein is the phloem-transported factor that initiates flower development in shoot meristems [4, 8, 16, 36, 37]. While flowering has been known to be sparse and delayed in cassava, it was not previously known whether this was due to deficiencies upstream or downstream of FT signal production. In another member of the Euphorbiaceae family, *Jatropha curcas*, an *FT* homolog was isolated, and when Jatropha plants were transformed with this gene under the control of the strong constitutive 35S-CaMV promotor, plants flowered extremely early [10], as expected for FT involvement. The current findings are also in agreement with studies in several species where overexpression of Arabidopsis *FT* induced earlier flowering. For example, in the late-flowering tree Eucalyptus, when atFT was driven by the 35S-CMV promotor plants flowered very early, within 1 to 5 months after transplanting [15]. Also, in apple trees, overexpression of Arabidopsis *FT* driven by 35S-CaMV promotor resulted in flower development directly from callus [16], and in poplar trees, atFT overexpression driven by a heat inducible promotor gave substantially earlier flowering [19]. Such studies, as well as the current investigation with cassava, indicate that the necessary components of the FT response system downstream of FT production are present and functional in the shoot apical meristems of these species, and that they are capable of interacting with the Arabidopsis FT gene-product to induce flowers much earlier than normal.

In cassava, branching occurs by outgrowth of axillary meristems subtending the shoot apical meristem (SAM), which results in two or more new shoot branches at the fork, occurs simultaneously with initiation of flower development at the original SAM [21, 27]. In the first tier of fork-type branching it is common in a large fraction of cassava genotypes for abortion of inflorescences and flowers such that these structures do not develop sufficiently to produce any mature flowers [27]. This was observed in the current study in the non-transformed genotype, 60444, which produced small flower stalks but did not produce any flower buds that exceeded the 2-mm diameter threshold for counting (Fig 3C). In striking contrast, all four atFT over-expression lines produced abundant, fully developed flowers (Figs 3C, 4 and 5B). Furthermore, flower production on inflorescences continued over a longer time-frame such that more flowers were produced and flowers at each tier had greater longevity before senescence (Fig 3D). Previous studies of *FT* overexpression have not reported this effect on flower prolificacy and longevity. Apparently cassava, with its limited flower development on the first-tier inflorescences, has revealed another effect of FT on enhancing the continued development of flowers that goes beyond floral initiation.

An additional effect of *FT* overexpression was shoot architectural alterations in the cassava atFT overexpression lines. In contrast with the absence of lateral branches in the non-transformed 60444 line, all lines overexpressing atFT produced abundant lateral branches, each of which forked and produced flowers (Fig 5A and 5B). This finding agrees with studies in which the overexpression of *FT* in cotton increased the extent of branching, apparently by altering the balance between *FT* and the flowering inhibitor, TFL [38]. Increased branching has also been reported in transgenic plants overexpressing FT in tobacco (*Nicotiana* spp.) [11] and Eucalyptus [15]. In contrast to flower initiation, flower prolificacy, and branching, flower and leaf organogenesis was not apparently affected by *FT* overexpression in cassava, as leaves and flowers were the same size and shape as in non-transformed plants (Fig 4). This agrees with the outcome in most reported studies, but contrasts with findings in *FT*-overexpressing lines of apple, which had more numerous petals, fewer stamens, and no pistils [16], and in *FT* overexpression lines of tobacco where there was also altered leaf morphology, increased leaf chlorophyll content and photosynthetic rates, and flower abscission [11].

In some plant systems that have vegetative storage organs, one or more *FT* homologs have been associated with stimulating the initiation and growth of these organs. For example, in onion, bulb formation is regulated by two antagonistic FT-like genes. AcFT1 promotes bulb formation, while AcFT4 prevents AcFT1 upregulation and inhibits bulbing in transgenic onions [39]. Another paralog, AcFT2 plays direct role in floral induction. Also, in potatoes (*Solanum tuberosum*), floral and tuberization transitions are controlled by two different *FT*-like paralogues [40, 41]. In the storage-root crop sugar beet, one *FT* homolog acts as a stimulator of flowering while a second *FT* homolog functions in repression of flowering [42, 43]. In *Jatropha curcas* and *Populus spp* (poplar), which are species closely related to cassava, JcFT plays an inductive role in flowering while the *Populus* paralogs PtFT1 and PtFT2 both function to induce flowering but also perform other roles associated with growth cessation, promotion of vegetative growth and bud set [10, 13, 44].

In the present study, we observed that the transgenic lines overexpressing the Arabidopsis *FT* in cassava showed reduced storage-root development as indicated by less storage-root dry weight per plant (Fig 6A) and fewer number of storage-roots per plant (Fig 6D). The transformants also had a smaller total plant size (Fig 6B), possibly because their increased development of flower primordia compromised the extent of new leaf production and hence restricted total plant growth. Alternatively, increased forking and axillary branch outgrowth and associated flowering in the atFT overexpression lines might have decreased production of leaves, which in turn affected whole-plant photosynthesis and growth. Studies have indicated that when branching is restricted, cassava storage-root yield is improved [45]. Moreover, the cassava atFT overexpression lines had a lower harvest index (fraction of total dry matter in storage-roots) (Fig 6C), indicating that rather than stimulating storage-root development, atFT might have had an inhibitory effect. Given that cassava is grown for storage-organ production, it is possible that domestication and breeding has led to genetic changes in FT that have the effect of increased storage-root production at the expense of flower development [46]. We hypothesize that cassava operates similarly to the species with vegetative storage organs discussed above, and may have regulatory pathways for floral development and storage-root development that are controlled by different *FT*-like genes.

We propose that this FT-expression system could be exploited to improve cassava breeding. Overexpression lines of cassava could be used as grafting partners, whereby the overexpression of atFT in understocks could provide a graft transmissible signal to scions of poor flowering lines. Graft-induced flowering with a profuse-flowering genotype as the understock has been used in other plant systems [7, 19, 47, 48], including cassava [21]. *FT* overexpression might serve as a particularly effective means of producing and delivering the flower-inducing signal from understocks to scions.

In conclusion, we have demonstrated that atFT overexpression in cassava hastens flower initiation, and increases lateral branching, similar to reports in other species. In addition, our findings provide the first report that in cassava, atFT overexpression substantially improves the prolificacy of flower production and the longevity of flower development. We also show that while cassava has the necessary signaling factors to respond to atFT such that flower development was enhanced, atFT did not stimulate storage-root development. These findings have the potential for furthering our understanding of flower development and for use in stimulating flower production in breeding.

## Supporting information

S1 Fig PCR of atFT in transgenic cassava and Arabidopsis genomic DNA. Lanes (left to right): cassava transgenic lines are labelled FT-02, FT-11, FT-13 and FT-17; No Template Control (NTC); non-transformed Arabidopsis Columbia ecotype (Col-0), and 60444 is the untransformed cassava plant. The amplification product size of atFT is 189 bp in the cassava transformants. Lane Col-0 is Arabidopsis Col-0 DNA; the * indicates the PCR product (1026 bp) of native FT including introns. Non-specific amplification products are labeled Φ. Lane M contains a 1kB ladder (Thermo Scientific GeneRuler 1kb Plus DNA Ladder).
(TIF)

S2 Fig Number of nodes between forking events in non‐transformed wildtype line (60444) and in four independent transformants. The number of shoot nodes between the soil surface and first forks, between the first-tier and second-tier forks, and between the second- and third-tier forks were counted at 5–6 months post planting in non‐transformed wildtype line (60444) and in four independent transformants treated with water and 1% ethanol respectively. Shown are the means ± SEM.
(TIF)

S3 Fig Total number of flowers per plant in water and ethanol treated control and transgenic plants. The number of flowers per plant were counted and recorded weekly, in non‐transformed wildtype line (60444) and in the four independent transformants treated with water and 1% ethanol respectively. Shown are the means ± SEM.
(TIF)

S4 Fig Time to start of flower senescence in water vs. ethanol treated transgenic plants and control. Flowering traits at each tier were recorded weekly to determine the time from flower appearance to initial date of flower senescence. Shown are the means ± SEM.
(TIF)

S5 Fig Harvest index in water vs. ethanol treated transgenic plants and control. Shown are the means ± SEM.
(TIF)

S6 Fig Storage-root dry weight in water vs. ethanol treated transgenic plants and control. Shown are the means ± SEM.
(TIF)

S7 Fig Total plant dry weight in water vs. ethanol treated transgenic plants and control. Shown are the means ± SEM.
(TIF)

S8 Fig Harvest index in water vs. ethanol treated transgenic plants and control. Shown are the means ± SEM.
(TIF)

S9 Fig Total number of flowers per plant on lateral branches. Data for plants treated with water and 1% ethanol were averaged. Shown are the means ± SEM.
(TIF)
