# Supplementary figures and images for: Overexpression of Arabidopsis FLOWERING LOCUS T (FT) gene improves floral development in cassava (Manihot esculenta, Crantz)
Source: PLoS One. 2017 Jul 28;12(7):e0181460. doi: 10.1371/journal.pone.0181460 (PMC5533431; doi:10.1371/journal.pone.0181460)

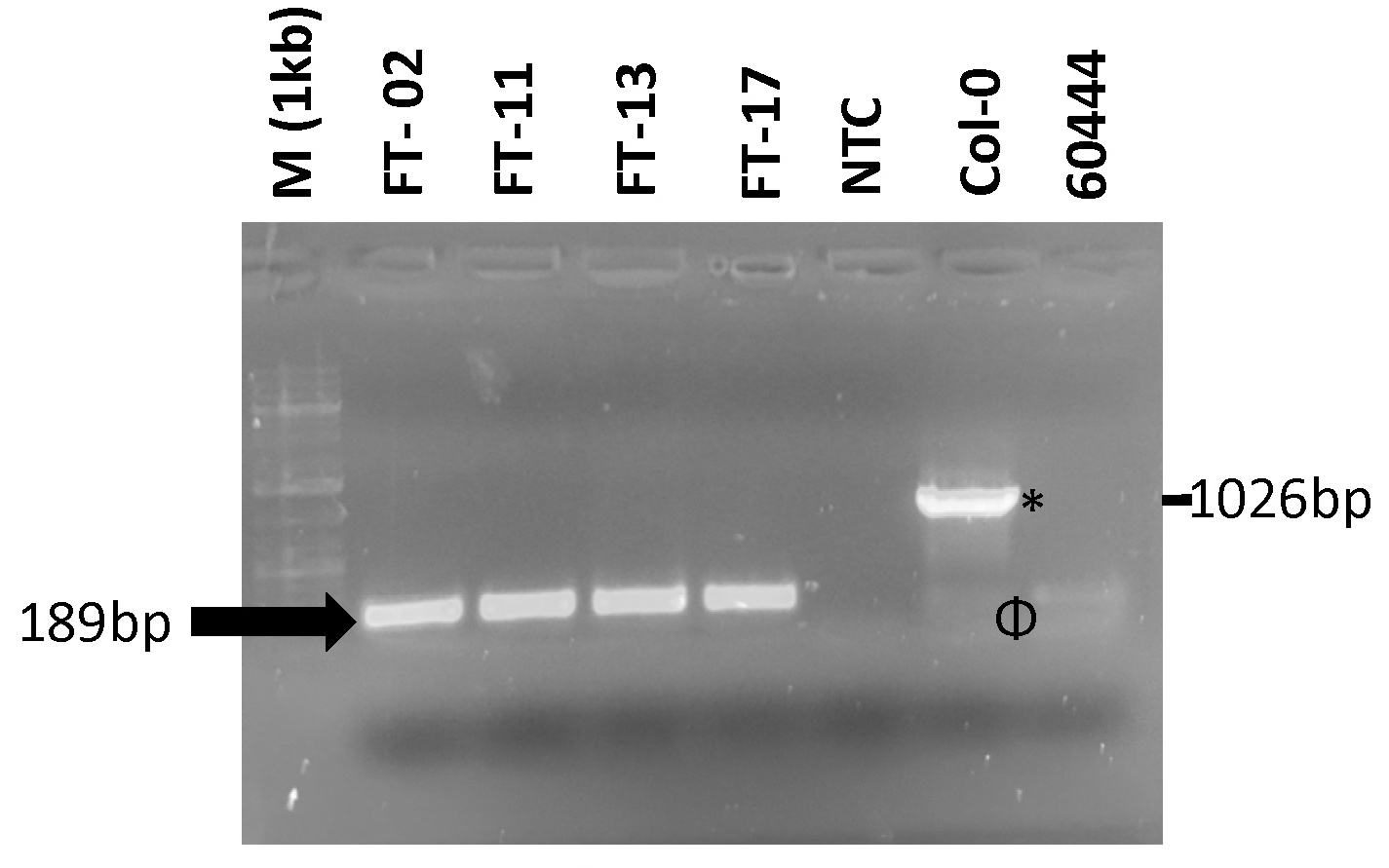

Supplement: S1 Fig — Lanes (left to right): cassava transgenic lines are labelled FT-02, FT-11, FT-13 and FT-17; No Template Control (NTC); non-transformed Arabidopsis Columbia ecotype (Col-0), and 60444 is the untransformed cassava plant. The amplification product size of atFT is 189 bp in the cassava transformants. Lane Col-0 is Arabidopsis Col-0 DNA; the * indicates the PCR product (1026 bp) of native FT including introns. Non-specific amplification products are labeled Φ. Lane M contains a 1kB ladder (Thermo Scientific GeneRuler 1kb Plus DNA Ladder). (TIF) [file pone.0181460.s001.tif]

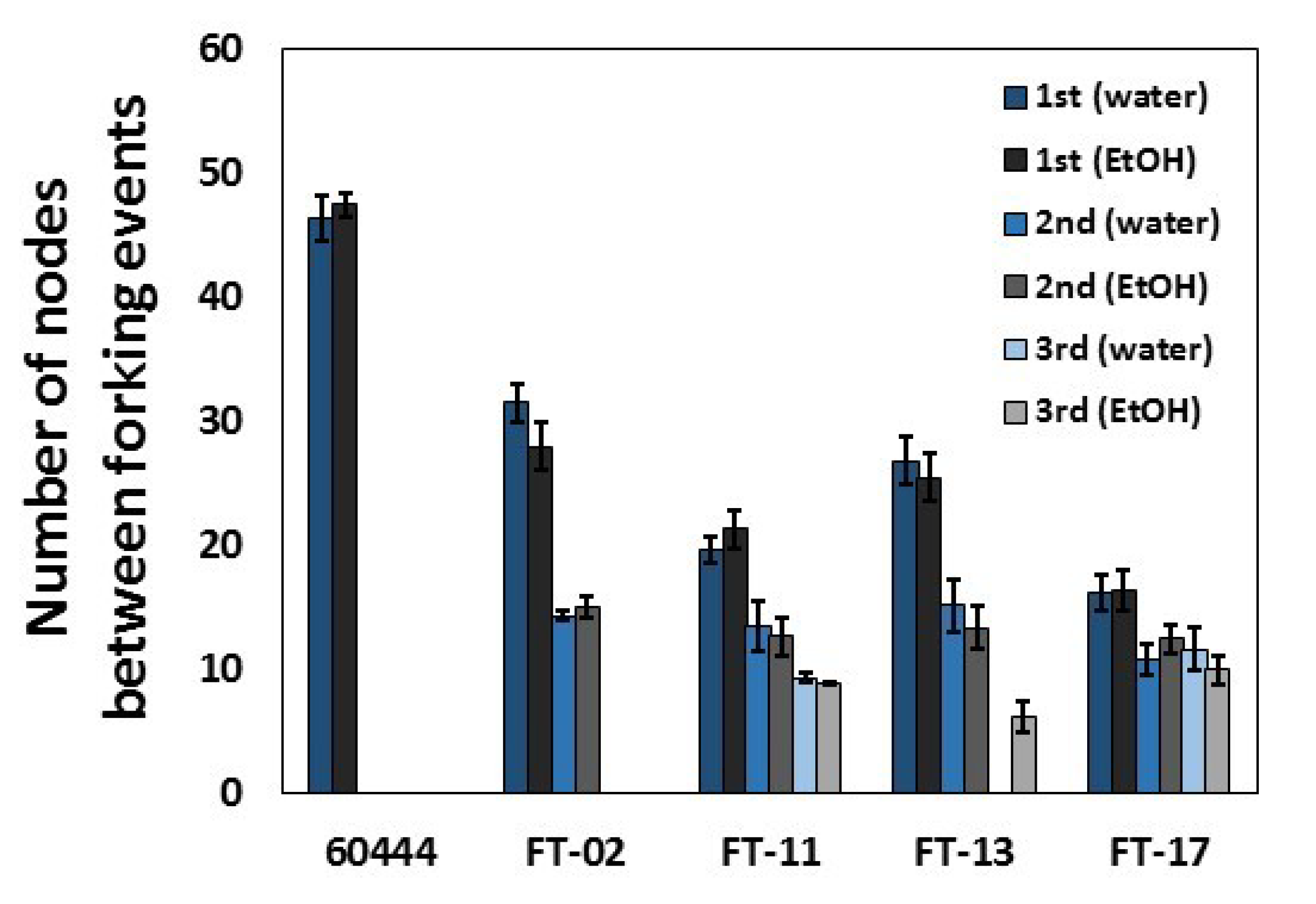

Supplement: S2 Fig — The number of shoot nodes between the soil surface and first forks, between the first-tier and second-tier forks, and between the second- and third-tier forks were counted at 5–6 months post planting in non‐transformed wildtype line (60444) and in four independent transformants treated with water and 1% ethanol respectively. Shown are the means ± SEM. (TIF) [file pone.0181460.s002.tif]

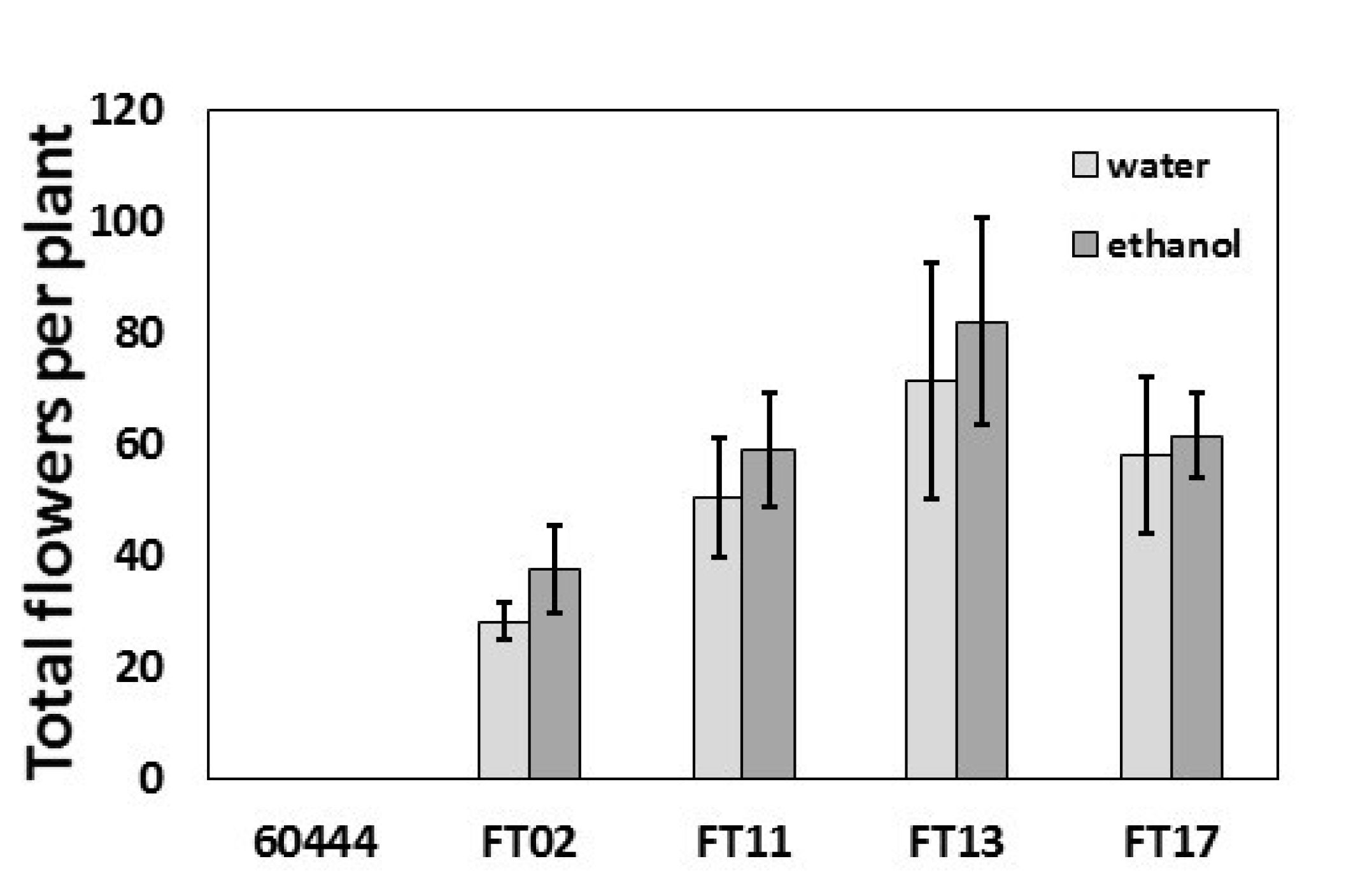

Supplement: S3 Fig — The number of flowers per plant were counted and recorded weekly, in non‐transformed wildtype line (60444) and in the four independent transformants treated with water and 1% ethanol respectively. Shown are the means ± SEM. (TIF) [file pone.0181460.s003.tif]

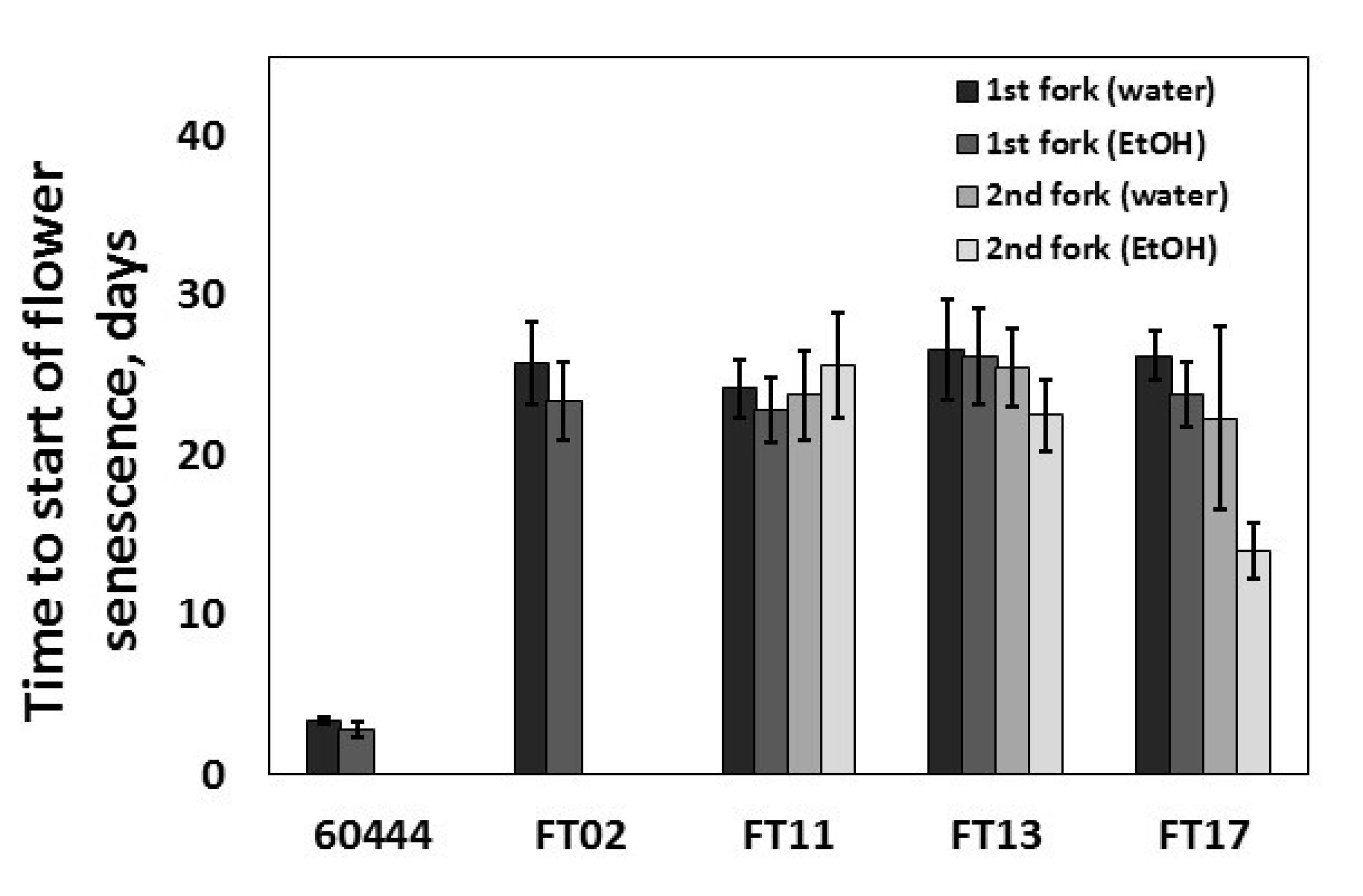

Supplement: S4 Fig — Flowering traits at each tier were recorded weekly to determine the time from flower appearance to initial date of flower senescence. Shown are the means ± SEM. (TIF) [file pone.0181460.s004.tif]

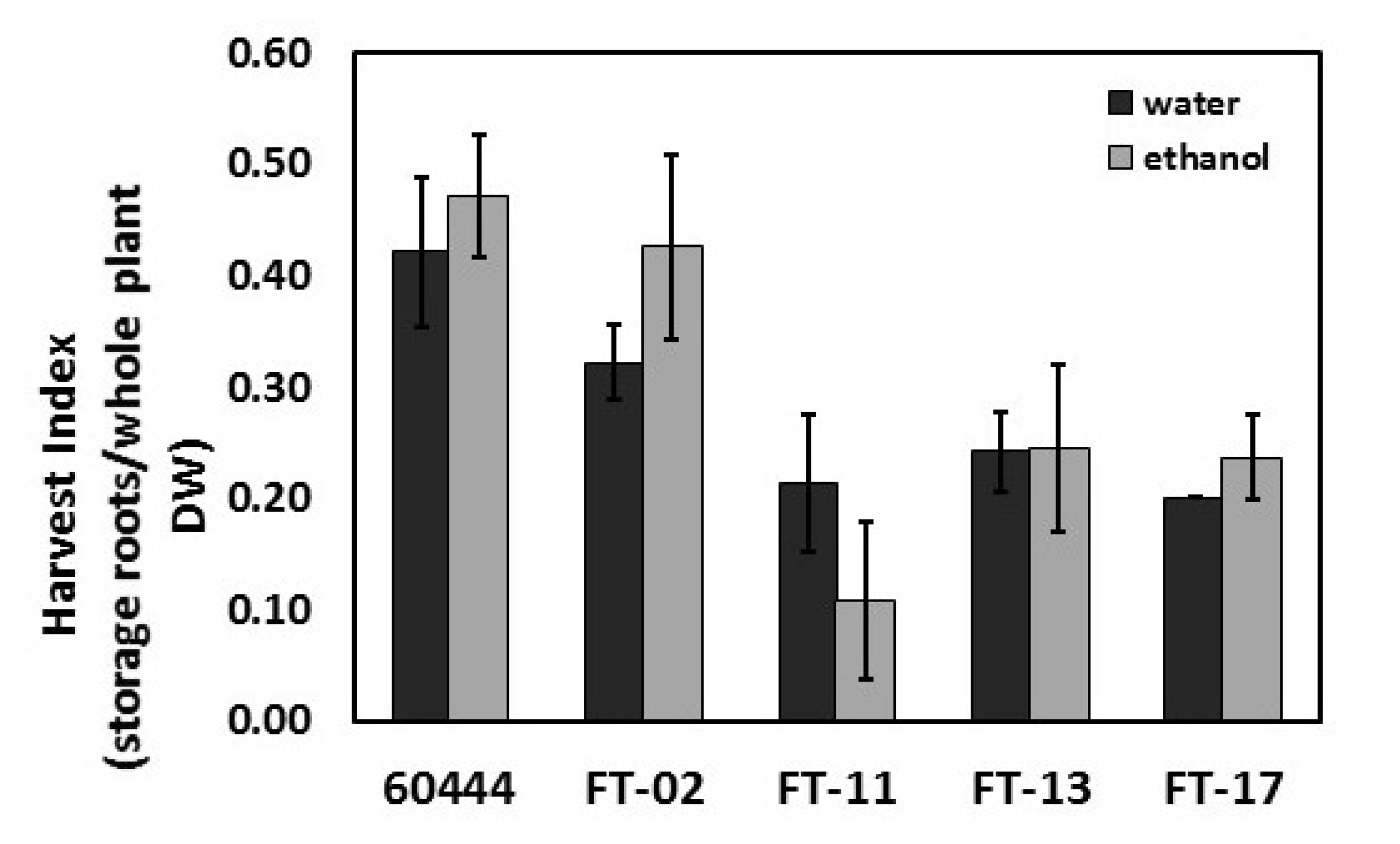

Supplement: S5 Fig — Shown are the means ± SEM. (TIF) [file pone.0181460.s005.tif]

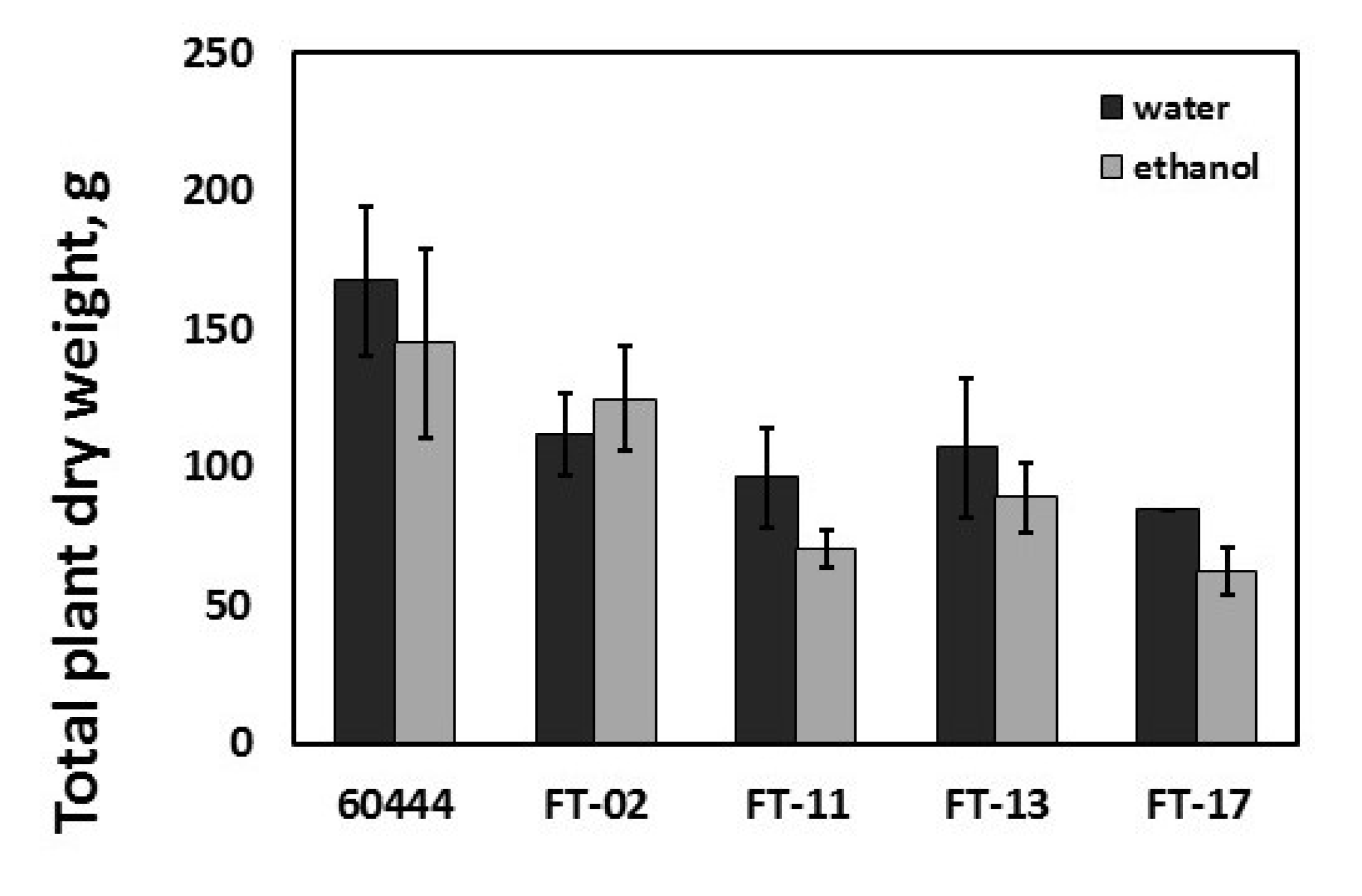

Supplement: S6 Fig — Shown are the means ± SEM. (TIF) [file pone.0181460.s006.tif]

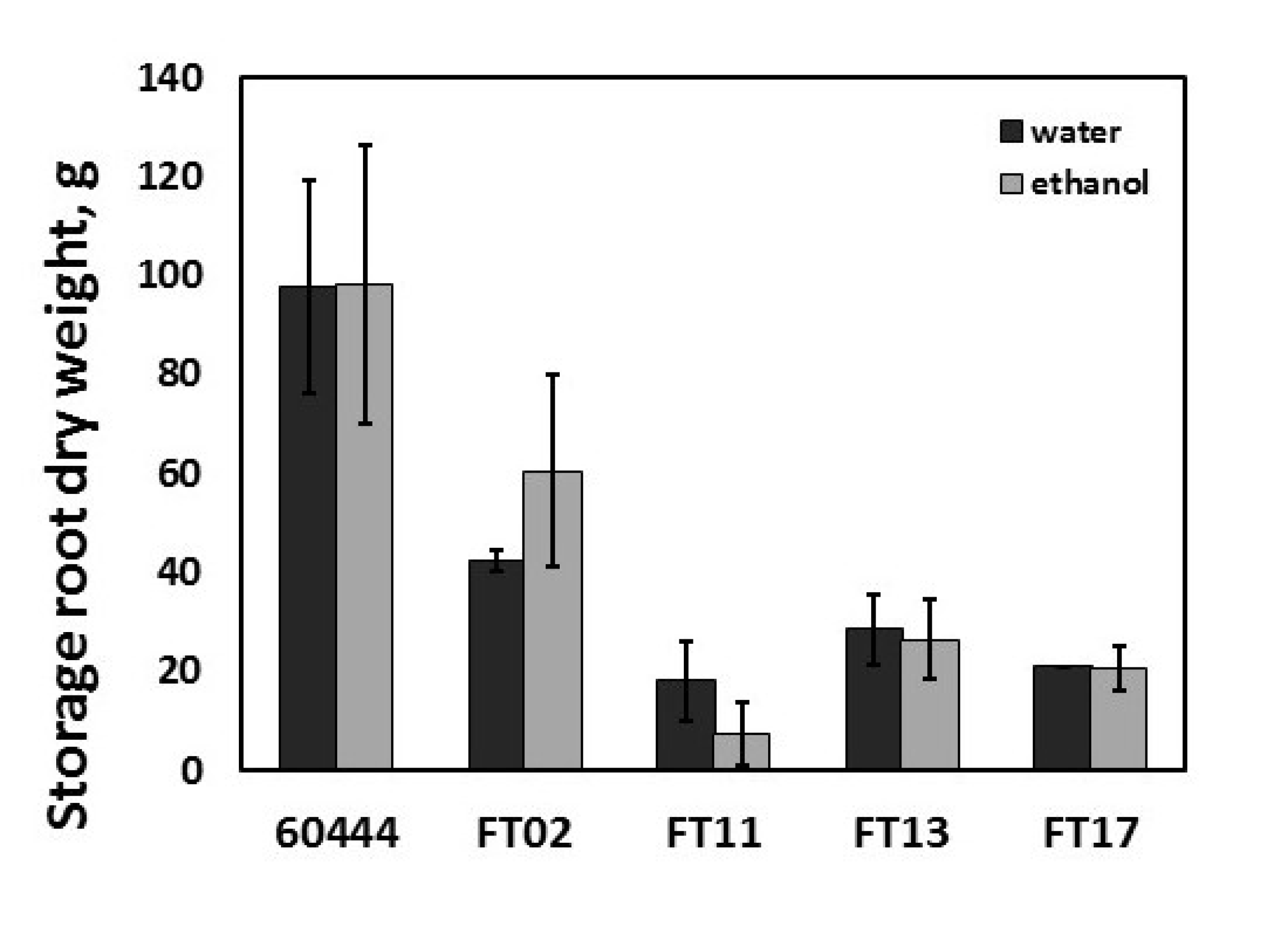

Supplement: S7 Fig — Shown are the means ± SEM. (TIF) [file pone.0181460.s007.tif]

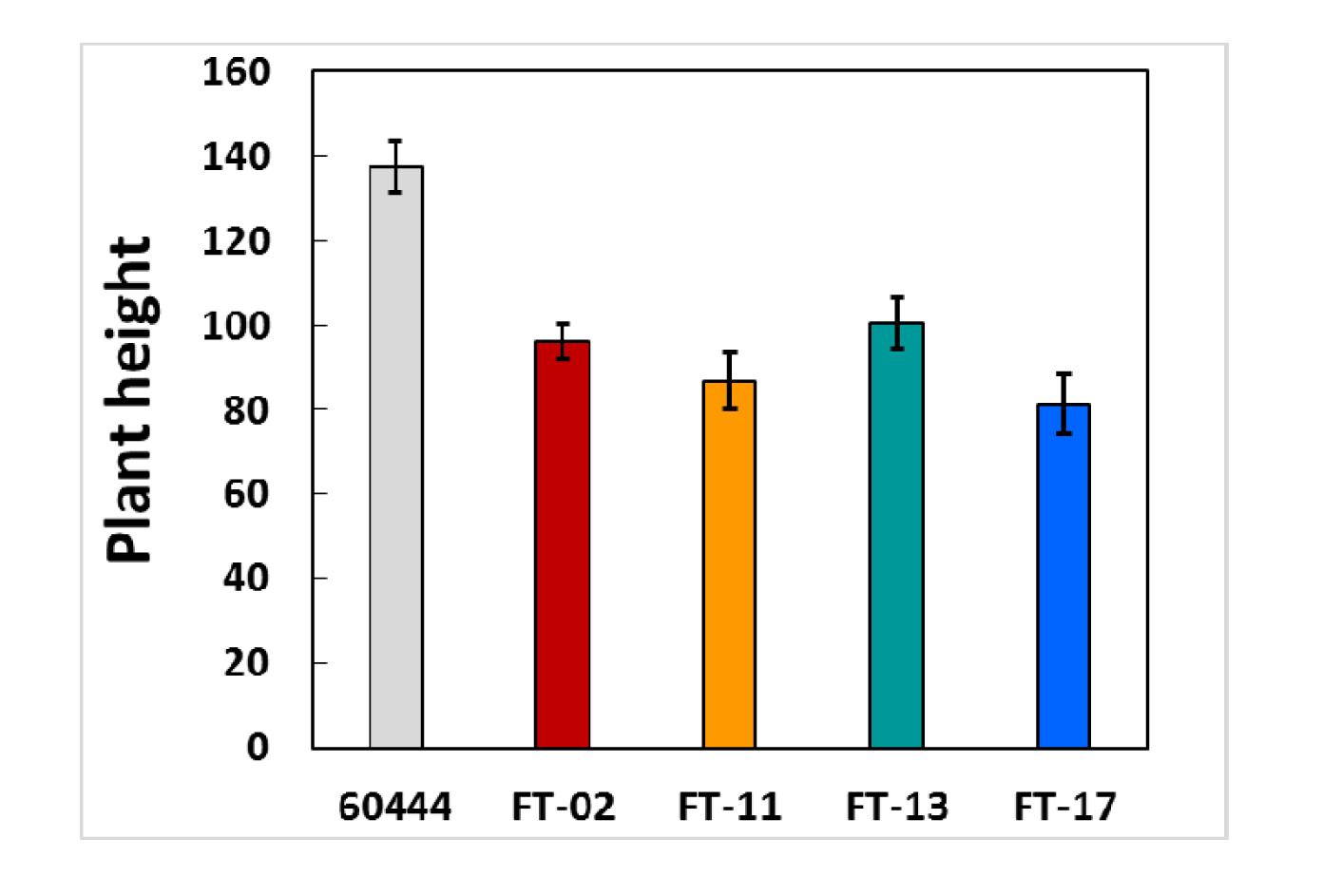

Supplement: S8 Fig — Shown are the means ± SEM. (TIF) [file pone.0181460.s008.tif]

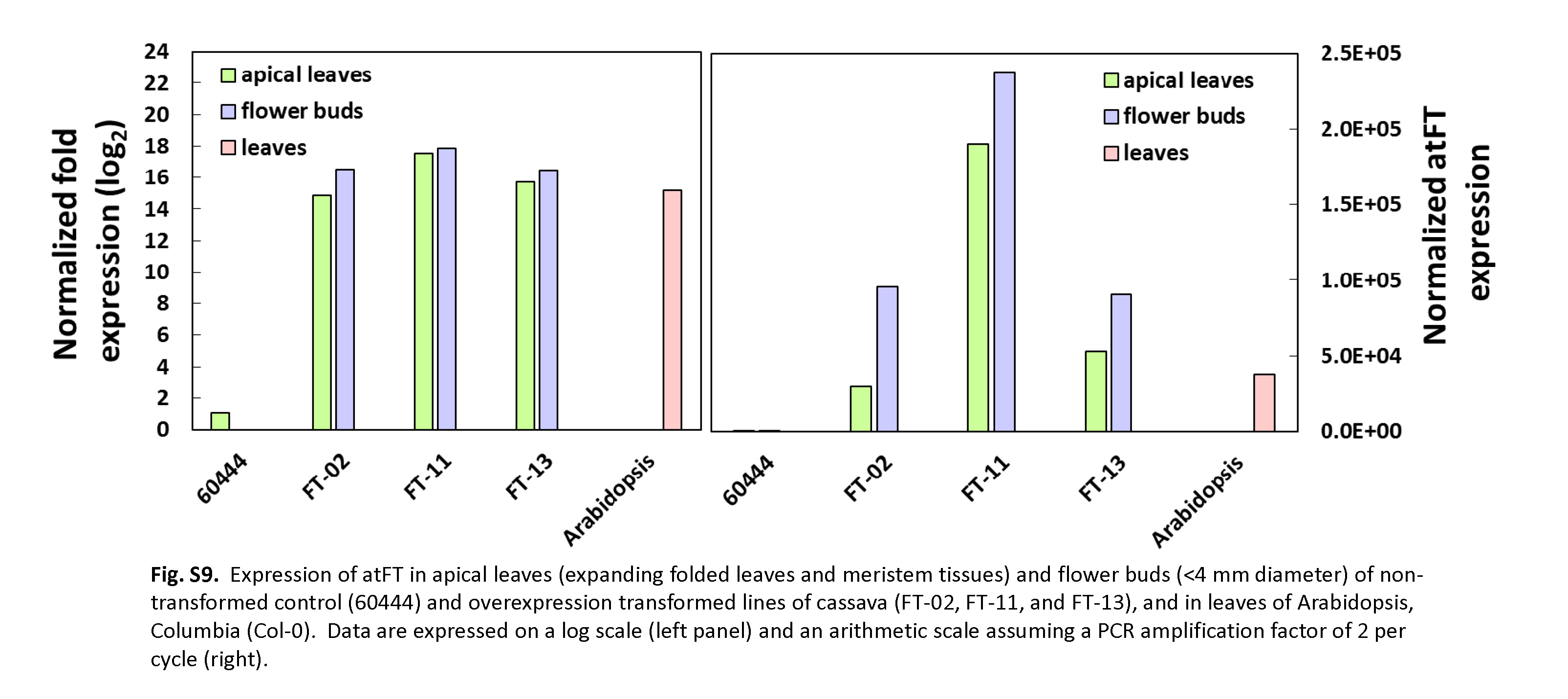

Supplement: S9 Fig — Data for plants treated with water and 1% ethanol were averaged. Shown are the means ± SEM. (TIF) [file pone.0181460.s009.tif]
